# Lymphocytic Choriomeningitis Virus Alters the Expression of Male Mouse Scent Proteins

**DOI:** 10.3390/v13061180

**Published:** 2021-06-21

**Authors:** Michael B. A. Oldstone, Brian C. Ware, Amanda Davidson, Mark C. Prescott, Robert J. Beynon, Jane L. Hurst

**Affiliations:** 1 Viral-Immunobiology Laboratory, Department of Immunology & Microbiology, The Scripps Research Institute, La Jolla, CA 92037, USA; brian.ware@cuanschutz.edu; 2 Mammalian Behaviour & Evolution Group, Leahurst Campus, Institute of Infection, Veterinary and Ecological Sciences, University of Liverpool, Neston CH64 7TE, UK; adave@liverpool.ac.uk; 3 Centre for Proteome Research, Institute of Systems, Molecular and Integrative Biology, University of Liverpool, Crown Street, Liverpool L69 7ZB, UK; markcprescott@icloud.com (M.C.P.); r.beynon@liverpool.ac.uk (R.J.B.)

**Keywords:** pheromones, MUPs, darcin, sex, virus, CTL, selection

## Abstract

Mature male mice produce a particularly high concentration of major urinary proteins (MUPs) in their scent marks that provide identity and status information to conspecifics. Darcin (MUP20) is inherently attractive to females and, by inducing rapid associative learning, leads to specific attraction to the individual male’s odour and location. Other polymorphic central MUPs, produced at much higher abundance, bind volatile ligands that are slowly released from a male’s scent marks, forming the male’s individual odour that females learn. Here, we show that infection of C57BL/6 males with LCMV WE variants (v2.2 or v54) alters MUP expression according to a male’s infection status and ability to clear the virus. MUP output is substantially reduced during acute adult infection with LCMV WE v2.2 and when males are persistently infected with LCMV WE v2.2 or v54. Infection differentially alters expression of darcin and, particularly, suppresses expression of a male’s central MUP signature. However, following clearance of acute v2.2 infection through a robust virus-specific CD8 cytotoxic T cell response that leads to immunity to the virus, males regain their normal mature male MUP pattern and exhibit enhanced MUP output by 30 days post-infection relative to uninfected controls. We discuss the likely impact of these changes in male MUP signals on female attraction and mate selection. As LCMV infection during pregnancy can substantially reduce embryo survival and lead to lifelong infection in surviving offspring, we speculate that females use LCMV-induced changes in MUP expression both to avoid direct infection from a male and to select mates able to develop immunity to local variants that will be inherited by their offspring.

## 1. Introduction

Mouse urine exhibits an obligate proteinuria, in the form of major urinary proteins (MUPs) [1,2,3,4]. These proteins, which account for over 99% of the urinary protein in a healthy male mouse, are synthesized in the liver and enter the urine via the glomerulus. MUPs are encoded by a multi gene complex on mouse chromosome 4, with at least 21 *Mup* genes that are transcriptionally active in the C57BL/6 laboratory mouse. Mouse MUPs form two subclasses: a cluster of “central” MUPs that exhibit a very high degree of sequence similarity and a smaller group of “peripheral” MUPs that each exhibit greater sequence divergence and are encoded by genes that flank the central MUP region. The major interest in MUPs stems from their biological activity in scent communication, as they provide both identity and status information about the donor animal [5,6,7,8,9,10,11,12,13,14].

Mice can detect MUPs directly on nasal contact with urine via specialized vomeronasal receptors [9]. MUPs also bind small volatile organic compounds (VOCs) in a central cavity with different affinities; the profile of expressed MUPs shapes an airborne odor profile as volatile ligands are slowly released and are detected through the main olfactory system [2,3,4,12]. One of the best defined MUPs is darcin (Uniprot: Q5FW60, MUP20_MOUSE), an 18,893 Da molecular weight protein that is expressed in adult male urine among wild house mice. Darcin acts as a sex pheromone that is responsible for female attraction to the male’s scent [15,16,17,18] and was named after Jane Austen’s romantic hero Fitzwilliam Darcy in the novel *Pride and Prejudice* [19]. Darcin also stimulates remembered attraction to the pheromone location and to the airborne odor signature that females associate with the pheromone, such that contact with darcin in a male’s scent results in strong female attraction to that specific male [12,18,20]. Most of the other MUPs in male urine are the products of the central region of the *Mup* cluster. These provide distinctive MUP signatures in genetically heterogeneous wild mice that are used for individual and kin recognition [5,7,12,21]. While mice of both sexes produce urinary MUPs, investment in these scent components is around 2–4 times higher in males than in females [6], although there is a slightly greater sex bias in the *Mus musculus musculus* subspecies compared to *M.m. domesticus* [8,13,14]. Some MUPs, such as darcin and central MUP7 (Uniprot: Q58EV3_MOUSE), are produced only by males, other central MUPs can show strongly male-biased expression, while some are expressed at similar levels by both sexes [22,23].

There is some plasticity in MUP expression [6,10,11,14,24,25,26], but, thus far, we know relatively little about how social and environmental events influence MUP production and the biological consequences of this. Disease status can be a major factor influencing an animal’s expenditure in sexual signals used in mate choice [27,28]. For example, darcin production by male house mice declines rapidly in response to immune challenge [10], although effects on the production of other MUPs have not been assessed.

Oldstone and Ware recently examined MUP production among male C57BL/6 laboratory mice infected with lymphocytic choriomeningitis virus (LCMV). They compared the effects of two variants cloned from the parental WE strain [29,30,31]. Variant 54 (identical to the parental WE strain) fails to generate virus-specific CD8 CTLs when inoculated into immunologically mature adult mice and leads to both a persistent infection and low MUP production in adult males [31]. By contrast, variant 2.2 (exhibiting a single amino acid change in glycoprotein residue 153) generates a robust CTL response that purges the virus and terminates the acute infection. MUP production is low during the acute infection phase, but greatly enhanced after viral clearance. There is no evidence of glomerular injury induced by infection of C57BL/6 mice with either variant, with no albumin present in urine samples during or following either infection. Instead, changes in urinary protein output specifically involve the production of MUPs [31]. As male mice invest heavily in MUPs in the scent signals they use to attract and compete for mates [6], changes in MUP expression with LCMV infection status and the ability of males to purge the virus may be signaled to females, altering a male’s attractiveness as a potential mate. For female mice, assessing the LCMV status of potential mates may have immediate importance as infection during gestation can substantially reduce offspring survival in utero and lead to lifelong infection in their surviving offspring [32,33]. As the ability to develop immunity to LCMV variants depends on genotype, mate selection based on male immunocompetence would allow females to pass on good genes to their offspring. Thus, understanding the specific effects of LCMV infection on MUP signaling could provide important insight into the evolution of sexual signals and whether male MUP signals provide reliable information that could allow females to avoid a virus that could seriously impact their reproductive success. However, while the Oldstone and Ware study detected broad changes in the total amount of MUP in urine samples, estimated from densitometry on Western blots [31], this was not able to provide a more detailed understanding of how infection and recovery from infection influence the expression of the different MUP isoforms that play key roles in sexual and competitive signaling [34].

Here we report the changes in urinary MUPs that occur among immunocompetent adult males in response to acute v2.2 infection. We also assess MUP changes when adult males were challenged with v54 leading to persistent infection, or after males that had been inoculated with v2.2 at birth were given virus-specific CTL by adoptive transfer in adulthood [35,36]. We show that LCMV WE v2.2 infection has differential effects on darcin, male-specific central MUPs and MUP10, which is expressed similarly in both sexes. We find that expression of darcin is strongly influenced by infection status, but suppression of a male’s central MUP identity signature is the strongest effect. However, clearance of acute infection by virus-specific CD8 CTLs, which results in lifelong immunity [31,33,35,36], leads to strong expression of darcin and the male’s signature MUPs. We discuss the potential impact of these changes for female sexual attraction and present a hypothetical model showing how females might use these signals to avoid LCMV infection in themselves and their offspring.

## 2. Materials and Methods

Mice and Viruses: C57BL/6 male mice (6–8 weeks old) and pregnant females were obtained from the rodent breeding colony at The Scripps Research Institute. Additional urine samples were also obtained from uninfected colony males for comparison (7–60 weeks old). All mice were maintained in pathogen-free conditions and handling conformed to requirements of the NIH, The Scripps Research Institute Institutional Animal Care and Use Committee (IACUC) and the Association for Assessment and Accreditation of Laboratory Animal Care (AAALAC). MUP and darcin studies utilized only males. Newborn mice were inoculated with 1 × 10^3^ PFU virus intracranially within 18 h following birth. Sexually mature males in late adolescence (7–8 weeks old) mice were injected with 2 × 10^6^ PFU virus intravenously [31]. Viruses used were WE strain and its variants 2.2 and 54. The origin of these viruses, their growth and quantitation have been reported [29,30,31]. Virus carried in blood and tissues were quantified in a plaque forming assay [29,30,31]. Assayed samples were diluted ten-fold and tested in triplicate.

CTL Assay: virus-specific CTL assay was performed as previously reported [31]. Days 7−8 post-infection were the timing used to measure acute CTL response. Deletion of CD8 CTL utilized monoclonal antibody YTS.1694 [31]. Deletion was 98%. Generation of memory CD8 CTL, their harvest and i.p. transfer of 2 × 10^7^ T cells to persistently infected mice have been reported [31,36].

Biochemical Assays: The virus overlay protein blot assay, Western blots and urine collections have been reported [31,37]. Protein assays, creatinine assays and electrospray ionization mass spectrometry (ESI-MS) to resolve different MUP phenotypes were performed as described [4,22,23,38,39,40]. For each urine sample, we calculated the total area of mass peaks in the ESI-MS spectra that corresponded to known MUP masses (range of 18,000–20,000 Da) and expressed each mass peak as a proportion of the total to examine changes in the relative proportion of different MUPs independent of total MUP output. Several MUPs that share very similar masses of 18,692–18,694 Da could not be distinguished in intact mass spectra (MUPs 1, 12, 2 and 15 and identical proteins 9, 11, 16, 18 and 19) and, thus, were analysed as a single group (MUP9^#^). To estimate the amount of each MUP mass expressed, the proportion of each mass peak was multiplied by the total urinary protein output [22].

Statistical Analysis: Data were analyzed using SPSS version 25 (IBM software). Groups were compared using ANOVAs, checking that residuals from each model approximated a normal distribution and log transforming variables where necessary to meet assumptions of parametric analyses. Repeated measures ANOVAs were used where samples were collected serially from the same individual males after CTL adoptive transfer. Bonferroni post-hoc comparisons were used to compare differences between multiple time points and a sequential Bonferroni adjustment was applied to significance levels across multiple MUP mass comparisons to correct appropriately for multiple comparisons.

## 3. Results

Earlier kinetic studies of response to LCMV WE v2.2 in sexually mature male mice [31] indicated that MUPs were present at low levels at days 5 or 10 post-infection but rose by day 20 and peaked at day 30. This strong MUP expression at day 30 post-v2.2 infection was maintained over the next 30 days of analysis. However, SDS-PAGE separation provides minimal resolution of different MUPs. Apart from a conformationally accelerated mobility of one MUP (darcin) and a slow migrating diffuse band of glycosylated MUPs, the majority of the abundant MUPs in urine migrate as a single, strongly staining band. The sequence similarity of the central MUPs makes individual identification by proteomics particularly challenging [4,22]. However, many of the MUPs have unique masses that are revealed by high resolution electrospray ionization mass spectrometry (ESI-MS) of intact MUPs in desalted urine samples. The ability to resolve several MUPs by mass offered an opportunity to explore the differential changes in expression of individual MUPs in response to viral infection and clearance. Here, we use ESI-MS to profile several MUP groups in urine during viral infection and after clearance. To allow assessment of changes in MUP output at a finer scale, we also determine total protein output corrected for variation in the dilution of urine samples by measurement of urinary creatinine [3,6].

### 3.1. Normal Male MUP Pattern and Output

**Figure 1:**
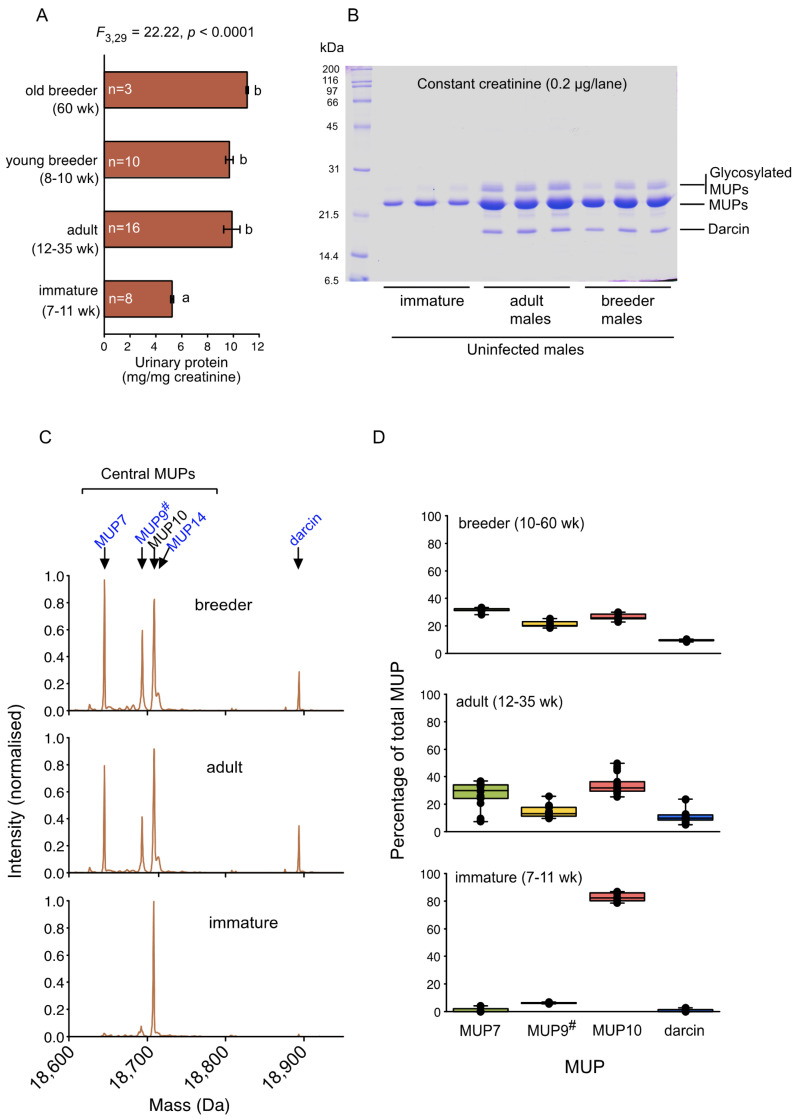
Urinary MUP output in urine of uninfected C57BL/6 male colony mice. (**A**) Total urinary protein output for a sample of immature and mature adult males housed in single-sex groups or with females for breeding. Protein is normalised to creatinine in urine to correct for variance in urine dilution. Different letters (a,b) indicate a significant difference between groups according to Bonferroni post-hoc comparisons (*p* < 0.0005). (**B**) Visualisation of urinary protein on reducing SDS PAGE, revealing the major MUP band, a high mobility darcin band, a slower mobility band of glycosylated MUPs and the absence of any other major protein bands in the urine samples (immature males aged 7 weeks; adult males aged 12, 19 or 26 weeks; breeder males aged 8, 10 or 60 weeks). (**C**) Resolution of MUPs by ESI-MS. The plots are deconvoluted spectra of the high abundance MUPs averaged over all urine samples shown in panel A for each type of male. Each peak represents the mature mass of a known MUP or is a coalescence of multiple MUPs with identical or very similar masses (MUP9^#^). (**D**) Expression of each of the major MUP peaks as a percentage of total MUP in each sample for immature, adult and breeding males as shown in panel A (median, 25th, 75th percentiles, full range and individual data points). MUPs 13, 14 and 17 are expressed at low level in this strain and are not shown. MUP9^#^ refers to the group of central MUPs with masses of 18692–18694 Da that could not be resolved (MUPs 1, 12, 2 and 15 and identical proteins 9, 11, 16, 18 and 19). MUP3 is glycosylated and requires alternative approaches for quantification that were not conducted in this study.

To establish a reference pattern, we first characterised the typical MUP output and profile of MUP isoforms expressed by uninfected males in the C57BL/6 local stock colony. Urinary protein levels of stock uninfected adult males were very typical of males of this strain [3,41], at around 10 mg/mg creatinine (Figure 1A). MUPs account for almost all of this high urinary protein output among healthy male mice (Figure 1B). Adult mice express a relatively fixed profile of different central MUP isoforms according to MUP genotype that varies between the sexes [23,42]. Uninfected colony males showed a MUP profile that is typical of C57BL/6 strain males (Figure 1C). In addition to a characteristic pattern of central MUPs, adult breeding and non-breeding male C57BL/6 also expressed darcin (MUP20; 10.51 ± 0.74% of total MUP, Figure 1D). However, young non-breeding males less than 12 weeks old had significantly lower MUP output (Figure 1A,B); these males express an immature MUP profile that consisted largely of MUP10 (Figure 1C,D). MUP10 is a central MUP that is produced at similar levels by both male and female C57BL/6 mice [22]. These immature non-breeding males did not express appreciable levels of MUP7, MUP9^#^ (a group of MUPs sharing similar mass that cannot be separated by ESI-MS), MUP14, or darcin, all of which are expressed at much higher levels in mature adult males under androgen control. Notably, though, 8–10-week-old colony males that were housed with females for breeding had very similar MUP output to older colony males (Figure 1A,B) and expressed a pattern of central MUPs and darcin that is typical of mature adult males (Figure 1C,D).

### 3.2. Clearance of LCMV Infection Leads to Enhanced MUP Output

Infection of 8-week-old C57BL/6 males with LCMV WE v2.2 initially leads to low MUP output over the first 10 days, but this increases significantly after males clear infection and peaks at day 30 post-infection (see Figure 3). We quantified the changes in MUP output among 18 matched pairs of singly housed littermate males where one male of each pair was recovering from v2.2 infection while its littermate control sib was singly housed at the same time without infection. Urine was sampled at day 20 and day 30 post-infection.

**Figure 2:**
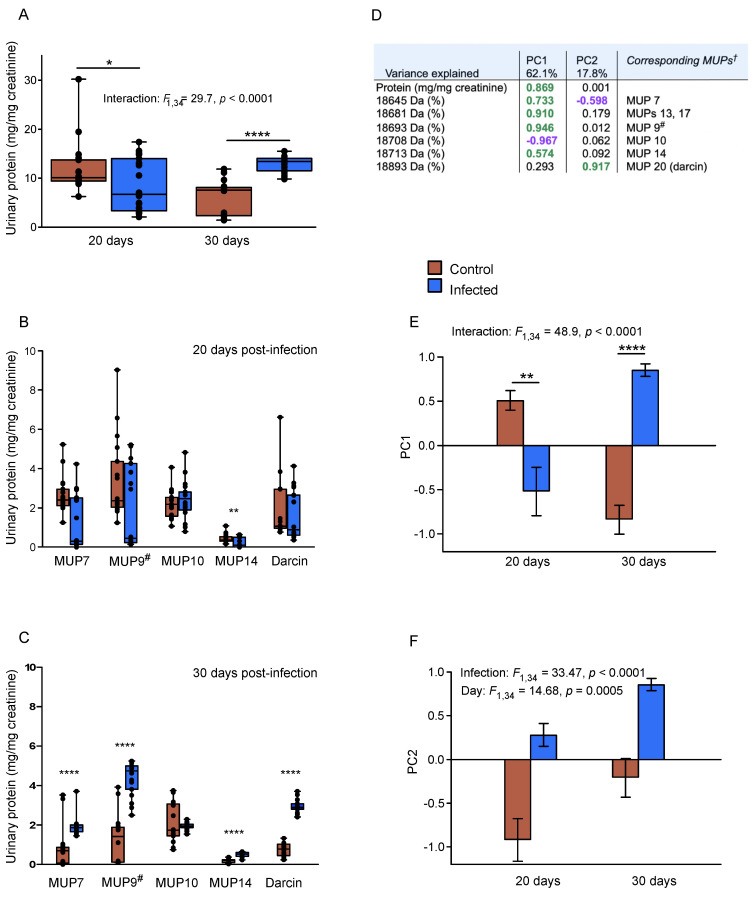
Changes in MUP output among adult males after acute LCMV v2.2 infection. Pairs of sib C57BL/6 males were singly housed at 8 weeks old and one male of each pair inoculated with v2.2 as previously described [31]. MUP output and profiles were analysed from urine samples taken at day 20 and day 30 post infection (N = 18 pairs per sample time point). Panel (**A**): total urinary protein output, corrected for urine dilution. Panels (**B**) and (**C**): amount of each major MUP peak expressed at day 20 or day 30 post infection, estimated from the total output and proportional area of each mass peak in the intact mass spectrum for each sample. Panels (**A**–**C**): plot medians, 25th, 75th percentiles, full range and individual data points. Panel (**D**): principal components analysis based on total protein output and proportion of each MUP mass, indicating loadings for each of the principal components derived (PC1 and PC2). Panels (**E**) and (**F**): PC1 and PC2 scores for infected and control males at day 20 and day 30 post-infection (mean ± s.e.m.). MUP9^#^ refers to the group of central MUPs of very similar or identical mass (see Figure 1). * *p* < 0.05, ** *p* < 0.01, **** *p* < 0.0001. Sequential Bonferroni correction was applied to analyses in panels (**B**) and (**C**) to correct for comparison of multiple MUP peaks.

Males recovering from v2.2 infection showed a significant increase in total MUP output between day 20 and day 30, while uninfected control littermates had high MUP output at day 20 (males aged 11 weeks) which reduced by day 30 (interaction between infection and time point, F_1,34_ = 29.7, *p* < 0.0001; Figure 2A). MUP output was very variable among infected males at day 20 post-infection, ranging from 2.1 to 17.4 mg protein/mg creatinine; approximately half the males had regained a level of MUP output similar to matched controls, but others still had low output (Figure 2A). However, by day 30, previously infected males consistently expressed a high level of MUP (range of 9.8–15.5 mg/mg creatinine), which was very similar to the level of output among control males at day 20 (Figure 2A). Surprisingly, though, the output of many of the uninfected sib control males declined to very low levels by day 30 (8/18 males), while others still had a fairly typical level of output for males of this strain.

To examine changes in the output of different MUPs, we used the area of each mass peak in the ESI-MS intact mass spectrum of each sample to estimate the proportional contribution of different MUPs to the total protein output. At day 20 post-infection, only MUP14 had significantly lower output in infected males compared to controls (Figure 2B), a male-specific MUP that has relatively low-level expression in C57BL/6 males [22]. Other MUP masses showed highly variable levels of expression between individuals, reflecting the variability in total protein output among both control and infected males at day 20. However, with the exception of MUP10, output of each MUP mass by day 30 was very significantly higher among males that had recovered from v2.2 infection compared to their uninfected control sibs (*p* < 0.0001; Figure 2C). The output of MUP10 did not change significantly with infection or between sample time points.

### 3.3. Enhancement of Adult Male MUP Pattern and Darcin

We used principal components analysis to examine the main patterns of variation in the total urinary protein output and the proportion of each MUP mass expressed. This derived two components with eigenvalues >1 that explained 79.9% of variance in the dataset. PC1 (62.1% of variance) reflected a strong contrast between the proportion of MUP10 expressed versus the total MUP output and the proportion of each of the other mass peaks in the male’s profile (Figure 2D). This reflected a consistent change in MUP profile with the total amount of MUP expressed: the higher the total MUP output, the lower the proportional contribution of MUP10 and the greater the proportion of MUPs that are expressed specifically by mature adult males (see Figure 1). Scores for PC1 showed a strong interaction between infection and time post-infection (Figure 2E). At day 20, males recovering from v2.2 infection still had lower MUP output due to much lower expression of male-biased central MUPs compared to control males. However, by day 30 males that had recovered from v2.2 infection had much stronger MUP output and expression of male-biased central MUPs than control males. Notably, though, the loading of darcin on PC1 was relatively low.

PC2 (17.8% of variance) was not influenced by total MUP output but instead reflected a strong contrast between the proportion of darcin versus the proportion of central MUP7 in a male’s MUP profile. Both darcin and MUP7 are expressed almost exclusively by mature adult males among both laboratory and wild house mice [11,14,23,24,43,44]. However, these two MUPs have distinct functions, with MUP7 contributing to the pattern of very similar central MUPs in mouse urine that provides individually distinctive odour signatures in wild mice, while darcin is the sex pheromone that induces inherent female attraction to these odour signatures [12,18]. PC2 scores were significantly higher in males recovering from v2.2 infection compared to their uninfected control sibs at both day 20 and day 30 (Figure 2F). Thus, males expressed a higher proportion of darcin compared to central MUP7 following v2.2 infection. There was also a general increase in the proportion of darcin to MUP7 between day 20 and day 30 in both infected and control males (Figure 2F), consistent with an age-related increase in the proportion of darcin relative to MUP7. As both the total MUP output and the proportional expression of darcin in the profile had greatly increased in males by day 30 post-infection, there was a particularly strong difference in the total amount of darcin expressed by males after recovery from v2.2 infection compared to matched control sibs (Figure 2C).

Variance in the extent to which males infected with v2.2 had recovered a normal male MUP output by day 20 was high (Figure 2A,B). We examined whether this was related to differences in the viral titer that males experienced during infection, or to how quickly males cleared the infection. Serum viral titers were sampled at days 3, 7, 10, 20 and 30 after v2.2 inoculation. Viral titers were highest at day 7, ranging from 8500 to 24,900 PFU/mL serum. All males had cleared infection by day 20, but 10/18 males still expressed a very low serum viral titer at day 10 (200–2000 PFU/mL). There was no significant relationship between serum viral titer at day 7 and the total amount of MUP expressed at either day 20 or day 30 post-infection. However, males with the highest viral loads at day 7 tended to express the lowest proportion of darcin at day 20 (*r*_17_ = −0.46, *p* = 0.053), although there was no difference by day 30 (*r*_17_ = 0.01, *p* = 0.97). Males that had cleared infection by day 10 also tended to have slightly higher MUP output by day 30, but this was not statistically significant (*F*_1,16_ = 3.46, *p* = 0.08) and early clearance of infection did not lead to more darcin expressed at either day 20 (*F*_1,16_ = 0.41, *p* = 0.53) or day 30 (*F*_1,16_ = 1.05, *p* = 0.32). Thus, we found only limited evidence that viral titer and speed of clearance influenced individual variation in how quickly males regained a normal mature male MUP expression, but all males had a fully developed mature male output by day 30.

### 3.4. Adoptive Transfer of CTL during Persistent Infection

**Figure 3:**
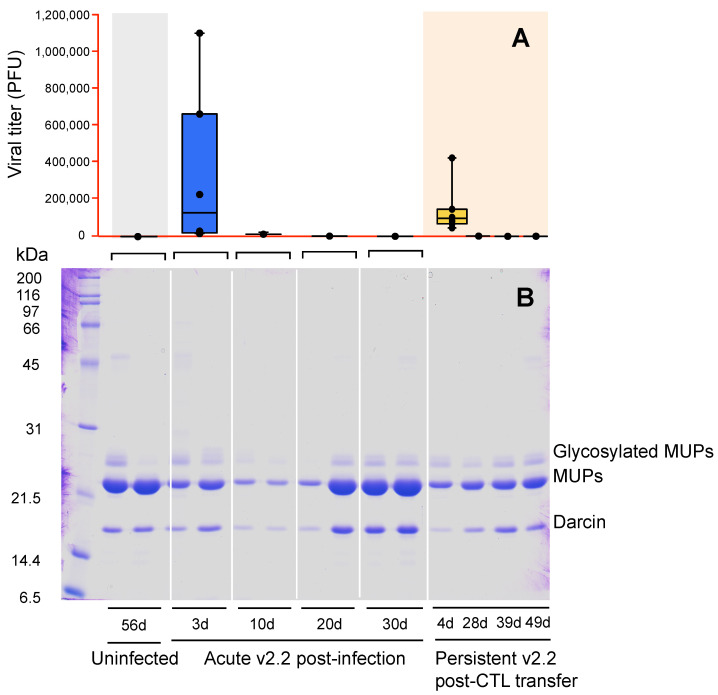
Typical urinary protein output during and after clearance of acute or persistent LCMV v2.2 infection. At 8 weeks of age, uninfected males were inoculated with v2.2 and blood and urine samples were analysed for viral titer (**A**) and urinary proteins ((**B**), MUPs resolved by SDS-PAGE) at days 3 or 7, 10, 20 or 30 (N = 6 males per time point). Another group of males (N = 7), inoculated with v2.2 within 18 h of birth to generate persistent infection, were treated with adoptive virus-specific CTL transfer at 8 weeks of age and sampled during infection and recovery.

Virus-specific CD8 CTLs play an essential role in the clearance of infectious virus and in the subsequent enhancement of MUP output [31]. Males with persistent infection, either from inoculation with v54 in adulthood or with v2.2 within 18 h of birth, lack a functional CD8 CTL response to the virus and have low adult MUP output. Importantly, providing virus-specific CTL by adoptive transfer to mice with persistent infection leads to viral clearance and a subsequent increase in MUP output [31]. Here we look at the changes in MUP output with time after adoptive CTL transfer in v2.2 persistently infected mice (inoculated within 18 h of birth) and compare this with output after adult infection with v2.2 (which stimulates a natural CD8 CTL response and recovery) or with v54 (no virus-specific CD8 CTL response leading to persistent infection) among males of matched age (Figure 3).

**Figure 4:**
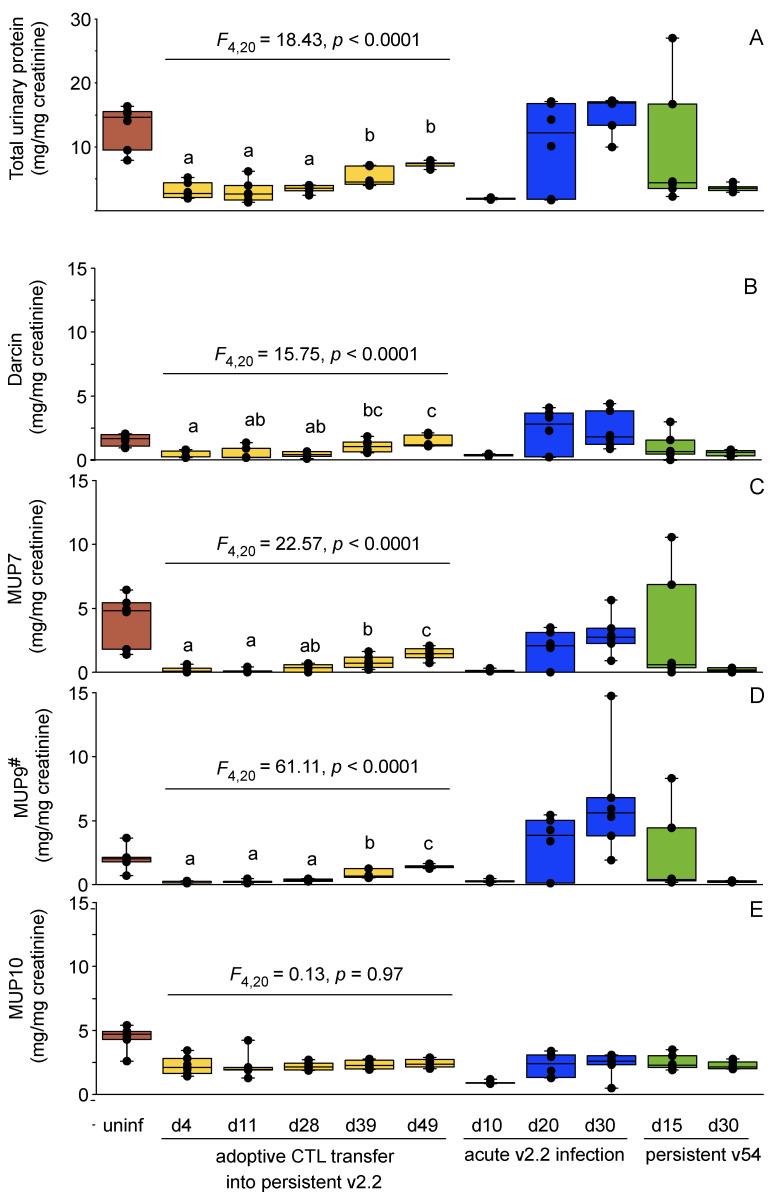
Changes in MUP output during and after CTL clearance of persistent or acute LCMV infection. Males with persistent LCMV v2.2 infection from birth (yellow) were tracked through viral clearance by adoptive CTL transfer (N = 7, see Figure 3 for viral titers). This was compared with males of the same age undergoing acute v2.2 infection and natural clearance (blue), inoculated with a persistent v54 infection (green) or uninfected (red) (N = 6 males per time point). (**A**): total urinary protein corrected for urine dilution. (**B**–**E**): amounts of each major mass peak (darcin, MUP7, MUP9^#^ and MUP10, respectively), corrected for urine dilution. Different letters above each column for adoptive CTL transfer experiment indicate time points that differ significantly using Bonferroni post-hoc comparisons (*p* < 0.05). MUP9^#^ is used to refer to the group of central MUPs of very similar or identical mass (see Figure 1).

Adoptive virus-specific CTL transfer into males with persistent v2.2 infection results in a substantial drop in viral titer by day 11 post-transfer, with no virus evident in serum at days 28, 39 and 49 (Figure 3A). Urinary protein output differed significantly between these time points (*F*_4,20_ = 18.43, *p* < 0.0001), due to a rise in MUP output that occurred between day 28 and day 39 post-transfer (Figure 3B and Figure 4A). The rise in MUP output following clearance in adulthood of a persistent v2.2 infection that was initiated at birth was slower than that observed after natural clearance of acute v2.2 infection that was initiated in adult mice. The output did not quite reach the same high level of MUP investment that is typical of healthy adult males of this strain (Figure 4A).

**Figure 5:**
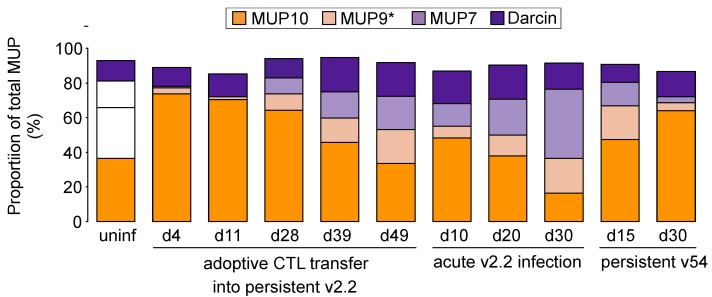
Proportional changes in MUP profile during LCMV infection. The MUP profiles from Figure 4 were expressed in relative terms, with each MUP mass displayed as a percentage of the contribution to total MUP output. Data are means for each treatment group and time point, sample sizes as in Figure 4. Uninfected males were of equivalent age and singly housed.

The pattern of central MUPs expressed by males during persistent v2.2 or v54 infection, or during an acute adult v2.2 infection (day 10), was similar to that of adolescent males that have an immature MUP pattern; the profile is dominated by MUP10 (non sex-specific) with very little expression of androgen-dependent central MUPs normally expressed by mature adult males (Figure 5). However, while adolescent males generally express very little darcin (Figure 1C,D), adult infected males retain darcin expression during infection, even when their total MUP output is very low (Figure 4B and Figure 5). Following adoptive CTL transfer into males with persistent v2.2 infection, the amount of MUP10 produced remained constant over all five time points sampled (*F*_4,20_ = 0.13, *p* = 0.97; Figure 4E), but expression of mature male central MUPs (MUP7, MUP9#) and darcin slowly increased (Figure 4B–D). By day 49 post-transfer, males expressed a profile of MUPs typical of healthy adult males (Figure 5).

Thus, persistent LCMV WE infection led to a dramatic reduction in MUP output that affected expression of all MUPs, but the strongest impact was on central MUPs that have strongly male-biased expression in healthy adult mice (MUP7, MUP9#; Figure 5). Output of central MUP10, which is not sex-specific in C57BL/6 mice, decreased during acute v2.2 viraemia, but otherwise was maintained at quite consistent levels of expression (Figure 4E). The proportion of darcin in each male’s MUP profile showed relatively limited variation across infection and recovery, with the amount expressed largely tracking infection-related changes in total MUP output, although the proportion of darcin was significantly elevated on recovery from infection and was lower in younger adult males.

## 4. Discussion

We had previously shown suppressed MUP output among adult C57BL/6 or FVB/N male mice that were unable to generate a virus-specific CD8 T cell response to LCMV WE v54 infection and were unable to clear the virus infection. By contrast, generation of a biologically active CTL response that cleared v2.2 infection led to a substantial increase in MUP investment which was greater than that of matched singly housed control males [31]. Here we report that such changes are not equal across all MUP isoforms, with infection differentially affecting the expression of the male pheromone darcin, central MUPs that have strongly male-biased expression and MUP10 that does not show sex-biased expression.

MUPs reflect a significant proportion of the protein synthesis in mouse liver and MUP mRNA may constitute as much as 5% of the total mRNA pool in male laboratory mice [45]. The irreversible loss of protein amounts to several 10s of milligrams a day for mature male mice, which imposes a significant energetic demand on the liver. Any changes that substantially influence the ability of this tissue to translate and secrete proteins are likely to impact on MUP output, such that MUP signals in mouse urine may reflect shifts in hepatic metabolic status. For example, calorie restriction can substantially diminish MUP production [46,47,48], probably mediated through growth hormone. Metabolic dysfunction in the diabetic obese mouse can also diminish production of specific MUPs (MUP1, [49]). Experimental models of murine schistosomiasis, which causes liver dysfunction and the disruption of major metabolic pathways, substantially diminish overall MUP production according to the severity of disease [50,51]. Male MUP output also declines with senescence, correlating with a decline in epididymal sperm counts and with reduced attractiveness of male urine signals to females [52]. Lopes and Konig [10] show that administering an immune challenge by injection of lipopolysaccharide reduces darcin production among captive bred wild males, as well as reducing male activity and ultrasonic signaling, although the production of other MUPs was not assessed. However, darcin output was much less diminished in mice parasitized with *Aspiculuris tetraptera* [53]. The overall picture is that there may be multiple levels of control on MUP output, reflecting overall energetic demand of their synthesis, mediation through altered endocrine signals, or effects that are specific to particular isoforms to elicit specific responses. However, very few studies have addressed how this differential control alters male MUP signals and the impact that this has on their functions in competitive scent signaling and female mate selection. 

Infection of adult males with LCMV WE v2.2 led to an early and substantial drop in male MUP production during acute infection, but males recovered expression of a normal mature male MUP pattern with consistently strong output by 30 days after the initial infection. This contrasted with a number of control sibs that showed a substantial decline in MUP investment after 30 days of single housing, including a major reduction in expression of androgen-dependent MUPs, such that their output resembled that of immature mice. This much reduced expression among some control males may have been a response to prolonged social isolation, which can induce a range of endocrine, brain and behavioural changes in mice, including an increased physiological response to stress, chronic activation of the HPA stress pathway and increased anxiety and depression-like behaviour [54]. However, in our wild house mouse colony, singly housed adult males that are regularly exposed to conspecific scents typically retain a normal mature male output of 10–30 mg MUP/mg creatinine [52,55]. The experience of competitive breeding conditions in enclosures stimulates males to increase their investment in MUPs even further [11,14,55], with outputs in some males rising to over 100 mg MUP/mg creatinine in semi-natural populations, although the level of investment varies widely between individuals (range of 10–113 mg/mg creatinine, [6]), with socially dominant males having the highest MUP outputs [11,14]. MUP output shows a similar wide range among males captured from the wild [8]. Under laboratory conditions, uninfected healthy males of most laboratory strains including C57BL/6 typically express around 10 mg MUP/mg creatinine [3,6,41], very similar to MUP levels expressed by uninfected mature males in the colony used in this study. After recovery from v2.2 infection, all males fully recovered a high level of MUP investment (mean of 13.8, range of 9.8–17.8 mg/mg creatinine), including relatively high levels of darcin and male-biased central MUPs. This suggests that mounting an immune response and clearance of v2.2 challenge enhanced subsequent outlay in MUPs, in contrast to the decline in MUP output among singly housed control males that were socially isolated for the same length of time.

Both darcin and central MUPs function together to provide an important male competitive sexual signal in mice. The effects of LCMV infection on these signals may help to explain why the prevalence of LCMV is relatively low in wild mouse populations despite both horizontal and vertical transmission of this virus. In nature, LCMV can be transmitted horizontally to conspecifics through contact with contaminated excretions (urine, faeces, saliva, tears, semen, milk). This usually leads to acute transient infection in naïve animals, which is cleared primarily by a virus-specific CD8^+^ cytotoxic T-cell response. However, most natural transmission is likely to be vertical, by in-utero transfer from mother to offspring, which leads to lifelong infection in the offspring [33,56]. Some LCMV WE variants, such as v54, can also induce persistent infection in adults by aborting the virus-specific CD8 T response [31,33]. As offspring of infected mothers become persistent infective carriers and naïve females can easily become infected via horizontal transmission through copious virus in excreta, we should expect the prevalence of LCMV in mouse populations to increase rapidly and reach an equilibrium close to 100%. However, surveys indicate that LCMV prevalence typically is relatively low among wild-caught mice. Approximately 9% of house mice were seropositive for LCMV in urban sites around Baltimore [57], 9.5% in a survey of farm and zoo populations in UK [58], 7% around the port of Yokohama [59] and 3.6% in a survey of house mice in Germany (cited in [57]). A number of mechanisms might contribute to reducing LCMV prevalence in mouse populations. For example, infected animals may have reduced survival under more challenging conditions outside of the laboratory. However, our findings suggest that LCMV infection is likely to have a substantial impact on female mate choice via effects on competitive male sexual signals. If this is the case, sexual signaling and mate choice could play a major role in limiting the spread of this pathogen in natural mouse populations.

**Figure 6:**
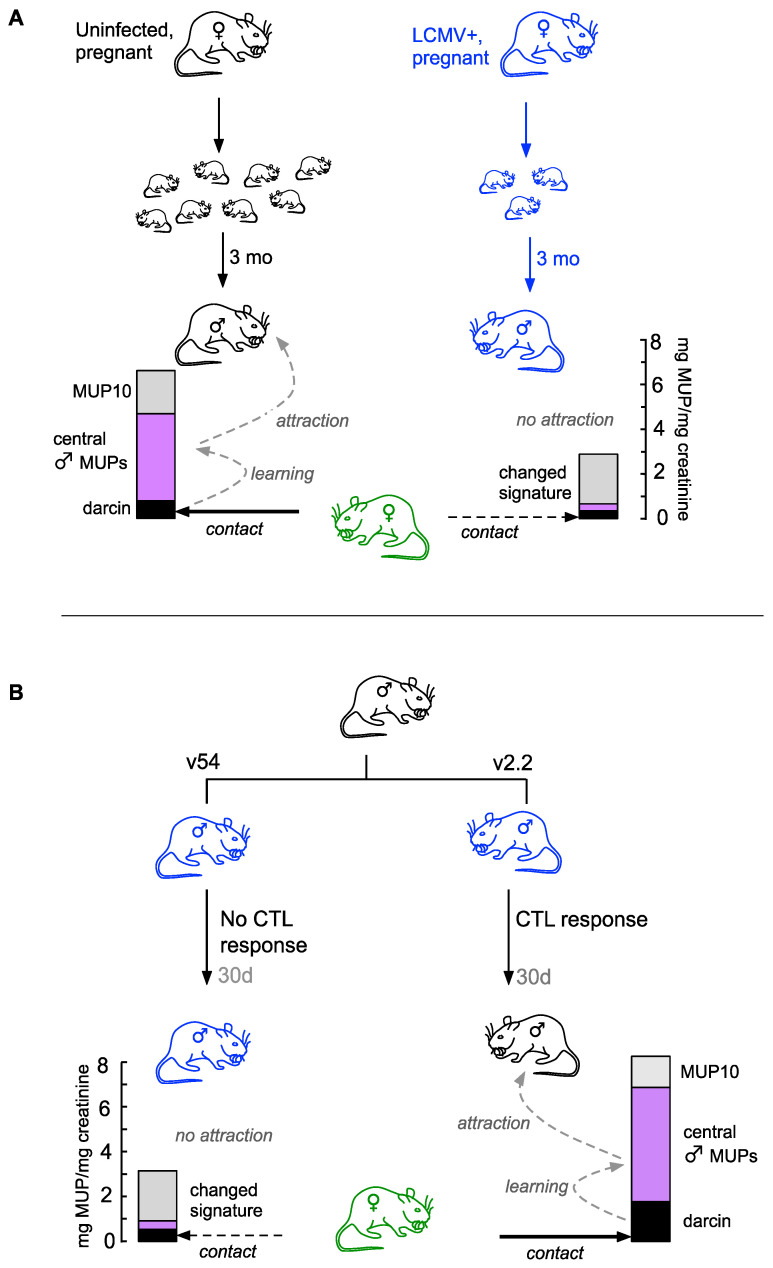
Effects of LCMV infection on male MUP expression and expected influence on female attraction. The cartoon depicts MUP signals produced by males that are infected with LCMV (blue mice) or uninfected/recovered from LCMV infection (black mice), with the predicted response of an adult female prospecting for a mate (green mice). Bars indicate the amount of darcin (black), male-biased central MUPs (purple) and central MUP10 (grey) produced by males of each type (data are mean mg MUP/mg creatine produced by singly housed C57BL/6 males in this study). (**A**) Fewer offspring survive from infected mothers and carry persistent infection with very weak male MUP signals when adult. Uninfected males produce much stronger MUP signals, including a central MUP signature and darcin, which stimulates females to contact scent and learn attraction to the individual owner. (**B**) Males that are able to clear LCMV infection gained in adulthood produce much stronger MUP signals including an individual signature and darcin, stimulating females to contact and learn attraction to the individual owner. See text for further details.

Our hypothesis is represented in Figure 6. Adult male house mice advertise their competitive ability to females through competitive scent signaling. This involves close interaction between the darcin sex pheromone (expressed by all mature males) and a male’s individual signature which is encoded by a set of polymorphic central MUPs that bind and slowly release a male’s airborne odour signature [12,38,60]. Males invest heavily in scent-marking their territories with urine that contains a strong darcin signal together with much more substantial investment in their individual-specific central MUP signature; with frequent refreshment, these scent marks continually release the male’s airborne signature. Females preferentially approach and investigate the strongest scent signals in the vicinity [38,61]. When females detect darcin on nasal contact with a male’s scent, the pheromone induces rapid learned attraction to the male’s associated odour signature, shaped by the male’s MUP profile [12,18]. They also learn attraction to spatial cues associated with the darcin pheromone location [15,20]. Subsequently, females evince a remembered attraction to that male’s odour and his location, which both focuses and amplifies female attraction to a male that can dominate and scent mark the local territory (Figure 6A). In the presence of females and competitors, males increase the strength and duration of their scent signals by increasing both their MUP output and rate of scent refreshment (e.g., [55]). 

However, MUP output is compromised in males with a persistent congenital LCMV infection gained from an infected mother. These males produce less darcin, although still at a level that could induce a positive female response [12,16]. Of greater significance, their total MUP output is substantially reduced, largely due to suppression of their central MUP signature (Figure 6A). This is likely to have several consequences for sexual signaling and for the infection risk that males pose to susceptible females. First, low MUP output will reduce the strength and duration of airborne odours released from urine marks that stimulate females to approach and contact a male’s scent [52]. This direct scent contact is essential for females to detect the non-volatile darcin pheromone and for females to learn any attraction to a male’s odour or location [18,20,62,63]. Secondly, even if females do contact and detect darcin in the male’s scent, suppression of the male’s polymorphic individual signature suggests that females will be unable to learn attraction to a specific individual odour associated with that male [12,18]. Thus, the changes in MUP production in persistently infected males predict that their scent signals will not stimulate normal sexual attraction from females. Male MUP signals are similarly compromised if susceptible uninfected males become infected after the perinatal period but are unable to generate an effective CTL response against a particular LCMV variant, again leading to persistent LCMV infection (Figure 6B). However, adult males that are able to mount an effective virus-specific CTL response and clear the virus subsequently produce strong MUP signals. After recovery from infection, males not only produce a relatively large amount of darcin, but they also invest particularly heavily in the central MUP signature that broadcasts their individual odour through scent marks. This will increase the likelihood that females will detect, investigate and learn attraction to the male’s individual scent signature and location (Figure 6B).

From a female’s perspective, an ability to mediate attraction to potential mates by using male sexual signals that are highly sensitive to LCMV infection status would reduce the likelihood that susceptible females will acquire infection from a mate. Mating and gestation are likely stages of vulnerability for female fitness. Laboratory studies suggest that maternal LCMV infection during early gestation, particularly in the first trimester, leads to high rates of abortion, foetal resorption and increased pup death through starvation [32,64], compromising the immediate survival and growth of pups. Our findings suggest that persistent infection among surviving pups also could greatly compromise the offspring’s future reproductive success. However, while our study has demonstrated clear effects of LCMV infection on male sexual signals in the laboratory, it should be acknowledged that the impact on reproductive success in natural populations is not yet known. In addition to the immediate infection risks for females seeking mates (a risk only for susceptible females), males vary in their ability to overcome horizontal LCMV infection and gain immunity according to both their genetic background and the specific LCMV variant [31,65]. To avoid passing genes to their offspring that may be ineffective in overcoming local LCMV variants, females should avoid infective mates that have not yet gained immunity, even if the female herself is not susceptible to infection transmitted by the male. This raises the intriguing possibility that both MUP and MHC responses to viral infection may interact in influencing female responses to male scent signals at this critical time. An MHC H-2 complex is essential for the generation of a virus-specific CD8 CTL response to acute viral infection to clear the virus. Between 15 and 20 days after CTL generation, the majority of CTLs are decreased to form virus-specific memory CTLs [66]. These memory CTLs respond more quickly, strongly and effectively in preventing reinfection with the same or a cross-reacting virus. Thus, once immune, males will be resistant to virus reinfection and provide females with little risk of infection through mating or through subsequent contact with the male or his excreta. However, when susceptible adult males become infected, viral loads increase before male MUP output is suppressed (see Figure 3, d3 post v2.2 infection, and Figure 4, d15 post v54 infection). Not only does this put susceptible females at risk if they have been attracted by scent from an apparently healthy male, but there will be no proof that the selected mate has the ability to overcome infection with that particular variant.

**Figure 7:**
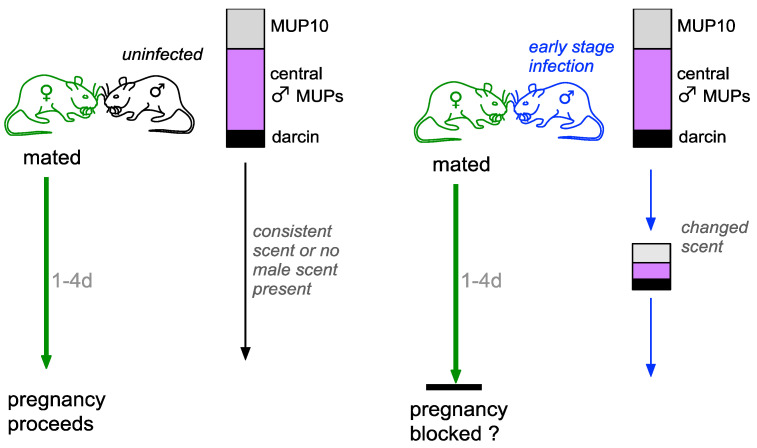
A putative model for the potential of LCMV to elicit pregnancy block (the Bruce effect). LCMV can be transmitted via semen and other excreta from infected mice. Females learn the scent signature of their mate in response to vaginocervical stimulation during mating. Males that are not infected with LCMV (black mouse) produce a strong and consistent signature of MUPs and associated ligands. Detection of consistent mate’s scent (or no male scent) over first 3 days post mating protects against implantation failure and pregnancy proceeds. Males at an early stage of LCMV infection during viral replication (blue mouse) initially produce strong MUP signals that rapidly become suppressed (also changing the profile of bound ligands). Exposure to changed scent of mate over first 4 days post mating in the absence of the remembered mate’s scent is predicted to inhibit prolactin release and leads to pregnancy failure. Bars indicate the amount of darcin (black), male-biased central MUPs (purple) and central MUP10 (grey) produced by males (data are mean mg MUP / mg creatine produced by singly housed C57BL/6 males in this study). See text for further details.

Here, we speculate whether changes in a male’s scent that are indicative of LCMV infection may have the potential to induce pregnancy failure, known as the Bruce effect, in recently mated females [67]. This would allow females to avoid investing in pups from sires that may not be able to develop LCMV immunity, as well as avoiding gestation if they are susceptible to LCMV infection. In laboratory experiments, females form a memory of the chemosignals of their mate specifically in response to vaginocervical stimulation during mating [68,69]. Over the next few days, exposure to chemosignals from an unfamiliar male in the absence of their familiar mate’s imprinted chemosignals inhibits prolactin surges in newly inseminated females that are needed to maintain luteal function during early pregnancy, resulting in pre-implantation pregnancy failure [70,71]. However, the presence of remembered chemosignals, either from the familiar mate, or from genetically identical males of the same strain, prevents the unfamiliar male scent from blocking the female’s pregnancy. The functional significance and evolutionary advantage of this response for females under natural conditions has been widely debated but remains enigmatic [70,72,73]. Newly mated females are sensitive to a variety of chemosignal differences compared to the imprinted mate’s scent, each of which can stimulate pregnancy block when manipulated separately on the mate’s background scent, including differences in MHC peptides in male urine [74], volatile odour signatures [75,76], low molecular weight ligands bound to MUPs [77], the level of the peptide ESP1 excreted in mouse tears [78] and polymorphic nonformylated NADH dehydrogenase peptides synthesised in mitochondria [79]. Studies of pregnancy block in mice so far have focused on chemosignal differences between different males, with the proposed explanation that females might block pregnancy if their mate disappears and is replaced by a foreign male, potentially allowing the female to remate with a better male or to avoid potential infanticide from the new male before making gestational investment [80,81,82]. However, some of these chemosignal components may also change within sires that undergo an acute LCMV infection during the critical period after a female has mated. Given the potentially very high costs to female reproductive success if mothers become infected with LCMV in early gestation, pregnancy block could be a highly effective strategy for mothers to delay investment in offspring until they have developed immunity. Further, as the ability of susceptible offspring to develop immunity to LCMV infection postnatally will depend on their inherited genotype, pregnancy block could prevent investment when it is unclear if sires can clear an acute infection. The suppression of a male’s individual MUP signature is very likely to change his profile of low molecular weight MUP ligands, such that females no longer recognise the male’s airborne odour [12]. As low molecular weight MUP ligands from an unfamiliar laboratory strain donor are highly effective in blocking embryo implantation [77], we speculate that this change in a male’s own individual signature may be sufficient to induce pregnancy block (Figure 7). Recently mated females are also highly sensitive to the presence of foreign MHC peptides in male urine [74,83], so it would be intriguing to test whether females can directly detect the presence of viral peptides in a male’s urine. However, other components of male chemosignals might also be particularly sensitive to LCMV infection as LCMV replicates not only in the liver, where MUPs are produced, but in many other tissues, including salivary and lacrimal glands [32,33,84]. Exocrine gland secreting peptide 1 (ESP1) is an androgen-dependent peptide that plays a key role in enhancing female receptive behaviour to allow successful copulation once females have selected a mate [85]. The peptide is produced in the extraorbital lacrimal glands by most wild-derived mouse strains and is released in male tears, although (similar to darcin) many domesticated laboratory strains do not express this peptide at functional levels. While we can only speculate that LCMV infection in lacrimal glands will alter expression of this key peptide, it is notable that pregnancy block occurs when newly mated females are exposed to males that excrete a different level of ESP1 to the male they mated with, in the absence of the mating male [78].

It remains to be tested whether females block pregnancy in response to the changing scent of a mate that is infective with LCMV. A number of factors make this an intriguing hypothesis. First, LCMV infection potentially has high short- and long-term costs for offspring (particularly for males) which could provide strong selection for preimplantation termination of pregnancy when females are at strong risk of infection in early gestation, or when a male’s infection becomes evident only after mating. Second, timing of the sensitive period for pregnancy block after mating (within the first 4 days) is appropriate to the likely development of scent changes in recently infected males. Third, males undergo substantial changes in scent that we might expect to stimulate pregnancy block based on experimental manipulations of scent components that have stimulated preimplantation failure in laboratory studies using healthy males. Lastly, pregnancy block does not occur if scent from the mating male remains present when females are exposed to scent from an unfamiliar male. This continued presence of an unchanged mate’s scent would allow females to recognise that the unfamiliar scent comes from a different male, whereas an unfamiliar scent that replaces the mate’s scent could signify a change in the familiar mate’s scent.

In conclusion, our findings suggest that MUP signalling in male mice is highly sensitive to LCMV WE infection and to the development of immunity through the generation of a virus-specific CD8 CTL response. Such T cells are generated after virus infects antigen presenting cells (primary dendritic cells). Dendritic cells process viral proteins into peptides that bind to the host’s MHC and are transported to the infected cell’s surface [33,86,87,88]. Given that MUP signals play key roles in female mate choice and attraction, it is tempting to speculate that the negative impact of LCMV on female reproductive success and that of their offspring may have contributed to the evolution of a sexual signaling system that is sensitive to a male’s infective status and ability to develop immunity to local variants of this virus. There has been a long-standing interest in the role of MHC in generating pheromones that influence mate choice ever since Boyse and colleagues used inbred mice and their H-2 congenic strains to demonstrate the ability of mice to discriminate between urine scents from strains that differ only in MHC type [89,90,91,92,93]. However, it has been more challenging to isolate MHC effects on a variable genetic background [94,95,96,97]. The roles that MHC-associated chemosignals play in mate choice have also proven controversial. For example, when differences in both MHC and MUP genotypes among wild mice are taken into account, females use the polymorphic profile of MUPs in male scent marks to recognise males but not their MHC-associated chemosignals [38], while mice mating freely in semi-natural enclosures avoid mates that share MUP genotype but not MHC [98]. By contrast, pregnancy block among laboratory mice is stimulated by MHC differences [74,76], by ESP1 expression [78] (encoded by a gene tightly linked to MHC on mouse chromosome 17, [5]) and by strain differences in low molecular weight ligands bound to MUPs [77]. We propose that further study of impact of LCMV and its variants on sexual signaling in mice might provide a test system to explain the relationship between MUP and MHC haplotypes, potentially reconciling one of the most notable longstanding debates in mouse semiochemistry.

## Data Availability

Data supporting reported results can be found in this manuscript.
